# Non-Image-Forming Light Driven Functions Are Preserved in a Mouse Model of Autosomal Dominant Optic Atrophy

**DOI:** 10.1371/journal.pone.0056350

**Published:** 2013-02-11

**Authors:** Georgia Perganta, Alun R. Barnard, Christiana Katti, Athanasios Vachtsevanos, Ron H. Douglas, Robert E. MacLaren, Marcela Votruba, Sumathi Sekaran

**Affiliations:** 1 Nuffield Department of Clinical Neurosciences, Nuffield Laboratory of Ophthalmology, University of Oxford, Oxford, United Kingdom; 2 Optometry and Visual Science, City University London, London, United Kingdom; 3 Moorfields Eye Hospital NHS Foundation Trust and NIHR Biomedical Research Centre, London, United Kingdom; 4 School of Optometry and Vision Sciences, Cardiff University, Cardiff, United Kingdom; Virginia Tech Carilion Research Institute, United States of America

## Abstract

Autosomal dominant optic atrophy (ADOA) is a slowly progressive optic neuropathy that has been associated with mutations of the *OPA1* gene. In patients, the disease primarily affects the retinal ganglion cells (RGCs) and causes optic nerve atrophy and visual loss. A subset of RGCs are intrinsically photosensitive, express the photopigment melanopsin and drive non-image-forming (NIF) visual functions including light driven circadian and sleep behaviours and the pupil light reflex. Given the RGC pathology in ADOA, disruption of NIF functions might be predicted. Interestingly in ADOA patients the pupil light reflex was preserved, although NIF behavioural outputs were not examined. The B6; C3-*Opa1* ^Q285STOP^ mouse model of ADOA displays optic nerve abnormalities, RGC dendropathy and functional visual disruption. We performed a comprehensive assessment of light driven NIF functions in this mouse model using wheel running activity monitoring, videotracking and pupillometry. *Opa1* mutant mice entrained their activity rhythm to the external light/dark cycle, suppressed their activity in response to acute light exposure at night, generated circadian phase shift responses to 480 nm and 525 nm pulses, demonstrated immobility-defined sleep induction following exposure to a brief light pulse at night and exhibited an intensity dependent pupil light reflex. There were no significant differences in any parameter tested relative to wildtype littermate controls. Furthermore, there was no significant difference in the number of melanopsin-expressing RGCs, cell morphology or melanopsin transcript levels between genotypes. Taken together, these findings suggest the preservation of NIF functions in *Opa1* mutants. The results provide support to growing evidence that the melanopsin-expressing RGCs are protected in mitochondrial optic neuropathies.

## Introduction

Autosomal dominant optic atrophy (ADOA) is the most common form of inherited optic neuropathy[1], [2], [3]. It is clinically characterized by a moderate to severe decrease in visual acuity, central visual field deficits, colour vision defects and temporal or diffuse optic nerve pallor[1], [3], [4]. ADOA is associated with a specific degeneration of retinal ganglion cells (RGCs)[4], [5]. Mutations in the optic atrophy 1 (*OPA1*) gene have been identified in most of patients with ADOA[6], [7]. The OPA1 protein is a dynamin-related guanosine triphosphatase (GTPase) anchored to the inner mitochondrial membrane.

Mouse models of *OPA1* ADOA have been generated to characterise further the pathophysiology of the disease[8], [9]. In the B6; C3-*Opa1* ^Q285STOP^ model, abnormalities in the optic nerve were detected by 9 months[8] without significant RGC loss[10]. Evidence of dendritic pruning localized to sublamina b of the retinal inner plexiform layer was observed from 10 months[10]. By 24 months, severe optic nerve degeneration was found[11]. Visual dysfunction has been described in this mouse model by both electrophysiological and behavioural testing. A specific deficit in the photopic negative response of the electroretinogram which reflects ganglion cell function, was detected in *Opa1* mutants from 12 months[12]. Importantly, deficits in the visual evoked potential (VEP) suggested light information was not being correctly relayed to the brain[12]. Concordantly, a reduction in visual acuity detectable by optokinetic drum screening was described[8].

A subset of RGCs is intrinsically photosensitive and expresses the photopigment melanopsin[13], [14], [15]. These cells are primarily responsible for providing the photic input to non-image-forming (NIF) behaviours. Intrinsically photosensitive RGCs are directly light sensitive but also act as conduits for rod and cone derived responses via dendritic inputs[16], [17], [18]. Ablation of the melanopsin expressing RGCs resulted in: (i) reduced entrainment to normal light/dark (LD) cycles[16], [17], [18], (ii) complete loss of circadian phase shift responses[17], (ii) absence of the suppression of locomotor activity to acute light exposure at night (‘negative masking’)[16], [18], iii) loss of the acute induction of sleep to brief light pulses[19] and iv) severe attenuation of the pupil light reflex (PLR)[16], [17]. These studies demonstrate that conventional RGCs also contribute to some NIF visual functions.

Although considerable research has been devoted to describing visual dysfunction in ADOA, NIF responses to light have not been extensively investigated. Given the pathophysiology of RGCs in *OPA1* ADOA patients and mouse models, deficits in light driven NIF functions might be predicted. Interestingly, the PLR is preserved in patients with *OPA1* ADOA[20], [21] although light driven circadian and sleep behaviours have not been assessed. We investigated circadian photoentrainment, negative masking, phase shift responses, acute light-induced sleep, the pupil light reflex and melanopsin expression in the B6; C3-*Opa1* ^Q285STOP^ mouse model of ADOA. The results support a preservation of the melanopsin RGCs and NIF light-driven functions in *Opa1* mutant mice.

## Methods

### Ethics Statement

All animal procedures were performed in accordance with the Animals (Scientific Procedures) Act 1986, UK. The protocol was approved by the University of Oxford Local Ethical Review Process (PPL 30/2812). All efforts were made to minimise suffering.

### Animals

Experiments were conducted on male heterozygous B6; C3-*Opa1* ^Q285STOP^ (*Opa1* ^+/−^) mice and age matched male littermate controls (*Opa1* ^+/+^). B6; C3-*Opa1* ^Q285STOP^ mice carry a heterozygous nonsense mutation in exon 8 of the *Opa1* gene (C-T transition at 1051 bp) resulting in protein truncation[8]. The mutation is embryonic lethal in homozygous animals. Food and water were available *ad libitum*.

### Circadian behaviour

Mice (*Opa1* ^+/+^: n = 6; *Opa1* ^+/−^: n = *7*; 11–13 months old) were individually placed in cages equipped with a steel running wheel and exposed to a 12∶12 light/dark (LD) cycle for 10 days. The light intensity used throughout (unless otherwise stated) was 200 lux (equivalent to ∼60 µW/cm^2^/s or 14.2 log quanta/cm^2^/s). Activity levels, the length of the active phase and period were calculated using ClockLab software (Actimetrics). A 3-h pulse of white fluorescent light (200 lux) was given at zeitgeber time (ZT) 14 (2 h after the lights were switched off on the night of day 10). The masking response was scored as the number of wheel revolutions during the light pulse expressed as a percentage of the number of revolutions made by the same animal during the same period on the previous night when there was no light pulse[22]. Total activity levels and activity levels in 1 h bins during the light pulse were also calculated. Animals were re-entrained to the normal LD cycle for 10 days.

Mice were subsequently released into constant darkness (DD) for 10 days. To induce phase shift responses animals were exposed to a 15-min monochromatic 480 nm or 525 nm (half peak bandwidth 10 nm) pulse of 1×10^11^ photons/s/cm^2^ irradiance at circadian time (CT) 16 (4 h after the onset of daily activity). Monochromatic light was applied in a specialised chamber with full internal reflectance using an LED light source (Honig Lichttechnik). In the control sham condition animals were handled in a similar way to the light pulse condition but maintained in darkness. There were at least 10 days between pulses. The magnitudes of the phase shifts were calculated using the difference between a regression line through steady state activity onsets prior to and after the pulse (ClockLab, Actimetrics). Transient responses in the first 2–3 days after the pulse were excluded.

### Sleep screening

Mice (*Opa1* ^+/+^: n = 5; *Opa1* ^+/−^: n = 6; 11–13 months old) were placed in cages equipped with infrared cameras for monitoring activity (Sentient Mini-night vision CCTV camera, Maplin, UK) and infrared LEDs. The output was recorded on a 16-channel digital hard-drive recorder (VXM4B-16, Videcon. PLC) at 25 frames per s with a resolution of 704×576 pixels. Animals were exposed to a 12∶12 LD cycle for 15 days. To determine the modulation of sleep behaviour by light, animals were exposed to a 1-h white light pulse (200 lux) at ZT14[23]. The immobility-defined sleep period for each mouse was assessed using video tracking software (ANY-maze, Stoelting). Sleep was defined as a period of immobility greater than 40 s. A previous study has confirmed that monitoring of immobility with videotracking has extremely high concordance (>95%) with EEG recordings for sleep determination[23]. Sleep latency (defined as the time between the onset of the light exposure and the first 2 min of continuous immobility) and total sleep, were also determined.

### Pupillometry

The right eyes of unanaesthetised mice (*Opa1* ^+/+^: n = 5; *Opa1* ^+/−^: n = 5; 24 months) were filmed under infrared illumination by a camera (Cohu, San Diego, USA) fitted with a zoom lens (giving a field of view of 8.7×6.9 mm), positioned in a plane parallel to the pupil. White light was delivered to the eye being filmed through a fibre optic (Leica CLS 100x). Since the action spectrum of wildtype mouse pupil constriction can be largely described by the mouse's scotopic sensitivity[24], measured spectral irradiances were weighted by the spectral absorbance of the murine rod visual pigment[25], as has been previously used[26]. Experiments were conducted during the light phase of the normal LD cycle to avoid any effects of circadian variation in the PLR[27]. After at least 1 h of dark adaptation, a light stimulus was delivered for 20 s. Animals were subjected to 14 different light intensities presented in ascending order of brightness with at least 2 min between stimulus presentations. The area of the pupil immediately before and 5 s after illumination was determined using Image J software. Pupil areas after illumination were expressed relative to the area of the pupil in darkness. Average irradiance/response curves were constructed for each genotype and fitted by a four term sigmoid relationship (SigmaPlot, Systat Software Inc., San Jose, USA).

### Immunohistochemistry

Animals were sacrificed at ZT8–ZT10 (*Opa1* ^+/+^: n = 3; *Opa1* ^+/−^: n = 3; 11–13 months old). Retinae were dissected and fixed in 4% paraformaldehyde (PFA) overnight. Retinae were incubated with an N-terminal polyclonal melanopsin antibody (PAS8331; rabbit anti-mouse (1∶500) in 1% Donkey serum, 0.1% Triton X-100 in PBS)[28]. Retinae were subsequently incubated with an Alexa Fluor 488 secondary antibody (donkey anti-rabbit IgG (1∶400) in 0.1% Triton X-100 in PBS; Invitrogen). A fluorescent microscope (Zeiss Axioskop, Germany) was used to view the retinal flatmounts and images were captured with a Hamamatsu Orca camera. Cell counts were performed using Adobe Photoshop software. Confocal images were captured using a laser scanning confocal microscope (Zeiss LSM510, Germany). Soma diameter was measured using Image J software (NIH, USA).

### Real-time Quantitative PCR

Animals were sacrificed at ZT8–ZT10 (*Opa1* ^+/+^: n = 5; *Opa1* ^+/−^: n = 5; 11–13 months old). RNA was extracted using the AllPrep RNA/Protein Kit (QIAGEN). Before RNA elution, on-column DNase treatment was performed. 500 µg RNA was reverse transcribed to cDNA with Superscript III Reverse Transcriptase (Invitrogen) using random hexamers as primers. Quantitative PCR (qPCR) was performed with Brilliant II Fast SYBR Green Master Mix. Transcript levels are expressed relative to the geometric mean of three house-keeping genes (GAPDH, B2M and PSMB2). The primers used were: melanopsin (*Opn4*) - Forward: TCACAGGGATGCTGGGCAATC, Reverse: TTCTTGTAGAGGCTGCTGGCAAAG and *Opa1* - Forward: TGACAAACTTAAGGAGGCTGTG, Reverse: CATTGTGCTGAATAACCCTCAA.

### Data Analysis

ANOVA or unpaired students t-tests were used to test for significance (p<0.05). All data are expressed as mean ± SEM. Light measurements were made with a lux meter (Macam Photometrics) or a spectrometer (Ocean Optics Inc).

## Results

### *Opa1* mutation has no effect on entrainment to a normal LD cycle

**Figure 1:**
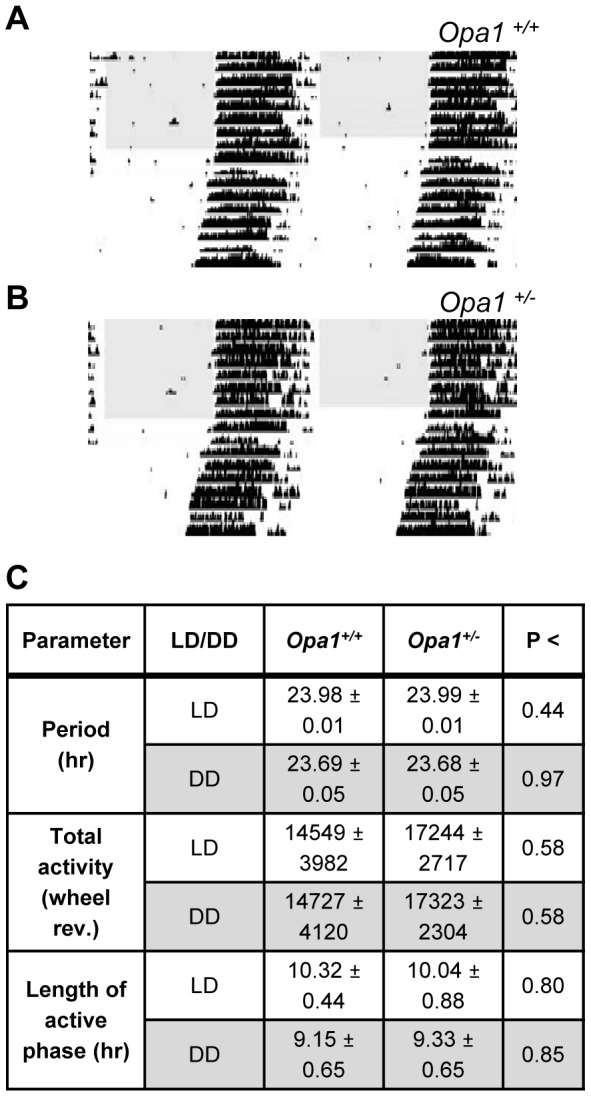
Circadian behaviour in *Opa1^+/+^* and *Opa1^+/−^* mice. Representative actograms from (A) *Opa1* ^+/+^ and (B) *Opa1* ^+/−^ mice entrained to a 12/12 LD cycle and subsequently released into constant darkness (DD). Each horizontal line corresponds to one day and the data has been double plotted. The black vertical bars represent activity (i.e. wheel revolutions). The shaded region represents lights ON. (C) Table showing average period (τ), total activity levels and length of the active phase in LD and in DD for *Opa1^+/+^* (n = 6) and *Opa1^+/−^* (n = 7) mice. There were no significant differences between genotypes (unpaired students t-test; p values are shown). All data are presented as mean ± SEM.

Circadian photoentrainment is driven by both melanopsin and conventional RGC pathways [16], [17], [18]. The ability of *Opa1* ^+/−^ and *Opa1* ^+/+^ mice to entrain to a 12∶12 LD cycle was assessed (Figure 1A,B). Both genotypes largely confined their activity to the dark phase suggesting the animals were entrained to the normal LD cycle. The period length (τ) and length of the active phase were equivalent for wildtype and heterozygous mice (Figure 1C). Total activity (average number of wheel revolutions per day) was slightly higher in *Opa1* ^+/−^ animals relative to the wildtype controls but this difference was not significant (Figure 1C). When released into constant darkness (DD), the phase of activity onset for the first day in DD was coincident with lights OFF of the previous LD cycle for both genotypes (Figure 1A,B) again suggesting that the mice were entrained to the light signal.

### Negative masking is preserved in *Opa1* ^+/−^ mice

**Figure 2:**
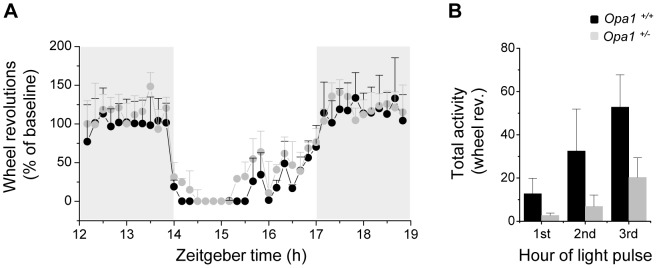
Masking response in *Opa1^+/+^* and *Opa1^+/−^* mice. (A) The average wheel running revolutions on the night (gray background) of the 3 h light pulse (white background) are plotted relative to the baseline levels (the night before the pulse) for *Opa1^+/+^* (n = 6) and *Opa1^+/−^* (n = 7) mice. The masking pulse completely suppressed activity in both genotypes immediately. ANOVA analysis found no significant effect of genotype on the baseline corrected activity levels (p = 0.468) (B) Hourly breakdown of activity during the masking pulse. A 2-way ANOVA using activity in each hour of light pulse and genotype as factors found a significant effect of hour of light pulse (p<0.005) but no significant effect of genotype (p = 0.143) and no interaction between genotype and light pulse hour (p = 0.359). All data are presented as mean ± SEM.

Previous studies have shown that light of ∼200 lux presented at night induced a complete negative masking response in wildtype mice[29] and in rodless/coneless mice[30] whereas negative masking was significantly impaired in melanopsin knockout mice[22]. We examined masking responses to 200 lux stimuli at ZT14 in *Opa1* ^+/+^ *and Opa1* ^+/−^ mice. Both genotypes significantly decreased their activity during the 3 h light exposure (data normalised to baseline from previous night; Figure 2A). *Opa1* ^+/+^ *and Opa1* ^+/−^ mice acutely stopped running during the first hour of the light pulse and activity levels subsequently increased, as has been previously shown in wildtype strains[22]. The baseline corrected activity levels during the light pulse was not significantly different between genotypes. An hourly breakdown of the number of wheel revolutions during the light pulse also showed no significant difference between genotypes (Figure 2B).

### Phase shift responses are maintained in *Opa1* ^+/*−*^ mice

Mice were released into constant darkness (DD) for a period of 10 days before application of phase shift light pulses. In DD, *Opa1* ^+/+^ and *Opa1* ^+/*−*^ mice displayed a wheel running activity rhythm with a period (τ) of ∼23.7 h. The average τ was almost identical between wildtype and heterozygous mice in constant darkness (Figure 1C). The total activity and length of the active phase were also not significantly different between genotypes in DD (Figure 1C).

**Figure 3:**
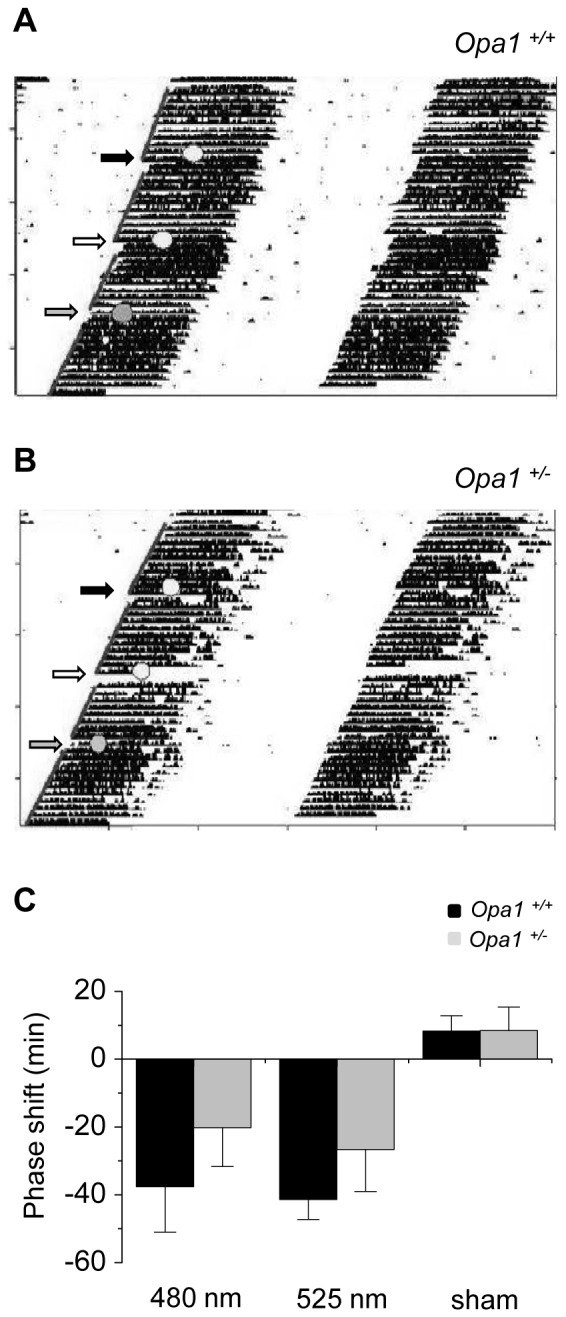
Phase shift behaviour in *Opa1^+/+^* and *Opa1^+/−^* mice. Representative actograms from (A) *Opa1* ^+/+^ and (B) *Opa1* ^+/*−*^ mice in constant dark (DD) conditions. Animals were exposed to 15 min light pulses every ∼15 days. Photon matched pulses at 480 nm (black arrow) or 525 nm (white arrow; 1×10^11^ photons/s/cm^2^) were applied at CT16. Animals were also exposed to a dark sham pulse condition (grey arrow). (C) The size of the phase shift response are plotted for the 525 nm, 480 nm and sham conditions for *Opa1^+/+^* (n = 6) and *Opa1^+/−^* (n = 7) mice. A two-way ANOVA with genotype and wavelength as factors was performed. There was no significant effect of wavelength (*p* = 0.66) or genotype (*p* = 0.17) and the interaction of genotype and wavelength was not significant (*p* = 0.91).

Light pulses of two different wavelengths were applied at CT16 to elicit phase shift behaviour. 480 nm light was applied to maximally stimulate the melanopsin-RGCs (λ_max_∼480 nm) and a 525 nm pulse was used to maximally stimulate the MW-sensitive cone (λ_max_∼508 nm) input to the melanopsin RGCs. The 480 nm pulse induced a delay in the phase of activity onset in both *Opa1* ^+/+^ and *Opa1* ^+/*−*^ mice (Figure 3A,B). The average magnitude of the phase shifts was not significantly different between genotypes (Figure 3C). In both genotypes, the period of the activity rhythm was not altered following the light pulse (τ after 480 nm pulse*: Opa1* ^+/+^ 23.6±0.09 h; *Opa1* ^+/*−*^ 23.6±0.07 h). The pulse of 525 nm also successfully induced a phase delay in activity onset in *Opa1* ^+/+^ and *Opa1* ^+/*−*^ mice (Figure 3A,B). There was no significant difference in the size of the phase shifts between genotypes (Figure 3C). Again the period length was not altered after the light pulse for each genotype (τ after 525 nm pulse*: Opa1* ^+/+^ 23.6±0.09 h; *Opa1* ^+/*−*^ 23.6±0.06 h). Importantly in a control sham pulse exposure, no obvious phase shift was observed in either wildtype or heterozygous animals (Figure 3C).

### Acute light induction of immobility-defined sleep is present in *Opa1* ^+/*−*^ mice

**Figure 4:**
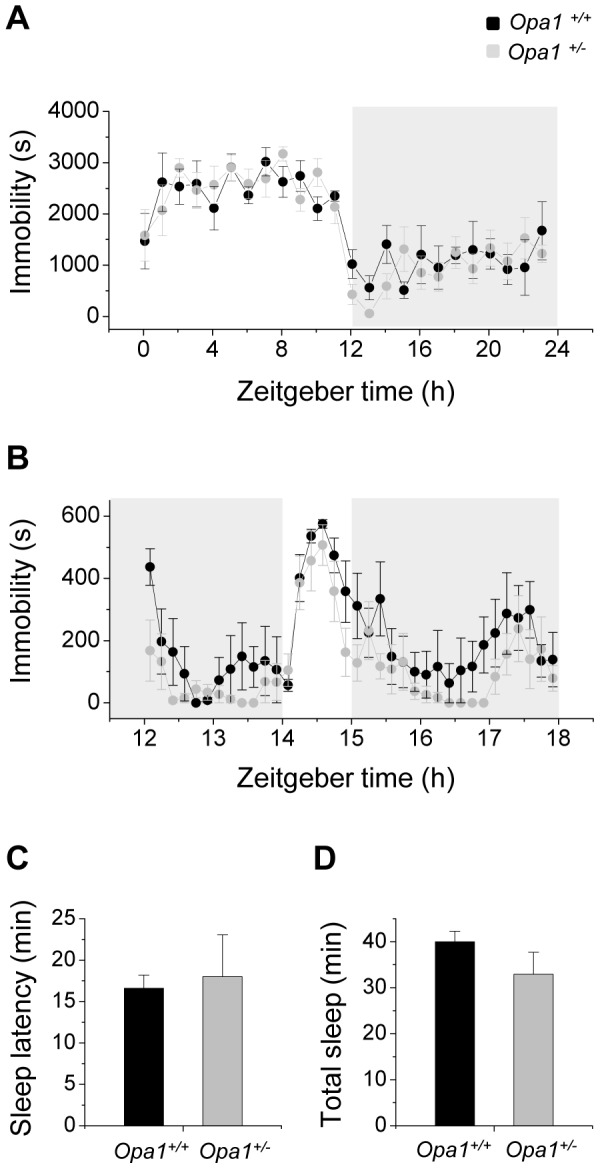
Induction of sleep by acute light in *Opa1^+/+^* and *Opa1^+/−^* mice. (A) The average immobility-defined sleep is plotted against zeitgeber time in a normal 12∶12 h LD cycle (1 h resolution) for *Opa1^+/+^* (n = 5) and *Opa1^+/−^* (n = 6) mice. Animals were largely immobile in the day phase of the LD cycle. White background indicates the day portion and grey background the night portion of the 24 h LD cycle. (B) The effect of the administration of the 1-h light pulse (white background) at ZT 14 during the night phase is shown. Both genotypes demonstrated an increase in immobility during the light pulse (10 min resolution). Quantification of (C) sleep latency and (D) total sleep during light exposure found no significant differences between genotypes *(*unpaired student's t-test). All data are presented as mean ± SEM.

Previous studies have shown that acute light exposure at night induces sleep in nocturnal species[19], [31], [32], [33] and that this process is distinct from the process of negative masking[19], [31]. At 200 lux, light induces sleep in wildtype mice and in rodless/coneless mice but sleep induction is impaired in melanopsin knockout mice[31]. We examined the acute effects of 200 lux light on sleep in *Opa1* mutant mice and wild-type controls by administering a 1 h light pulse 2 h after the onset of dark (ZT 14) when the homeostatic and circadian drives for wakefulness are high. We used videotracking assessment of immobility as a measure of sleep which has been found to highly correlate with EEG/EMG recordings of sleep[23]. *Opa1^+/+^* and *Opa1^+/−^* mice exhibited low immobility-defined sleep (i.e. high activity) during the dark period of a normal 12∶12 LD cycle as would be expected for a nocturnal species (Figure 4A). In response to the light pulse, a sharp increase in immobility-defined sleep was observed in both genotypes within 30 min of lights on (Figure 4B). Immobility levels subsequently declined to ∼50% of baseline by the end of the light pulse. The sleep induction response differed from negative masking where the suppression in activity levels was maintained during the entire first hour of the light pulse. This adds further support to acute light induced sleep and negative masking being distinct processes. The latency to the first 2 min of continuous immobility (sleep latency) was not significantly different between genotypes (Figure 4C). During acute light exposure, the total time spent asleep was also not significantly different between wildtype and mutant mice (Figure 4D) suggesting that both genotypes sustained the inhibitory response to light.

### Pupil light reflex is preserved in *Opa1* ^+/*−*^ mice

**Figure 5:**
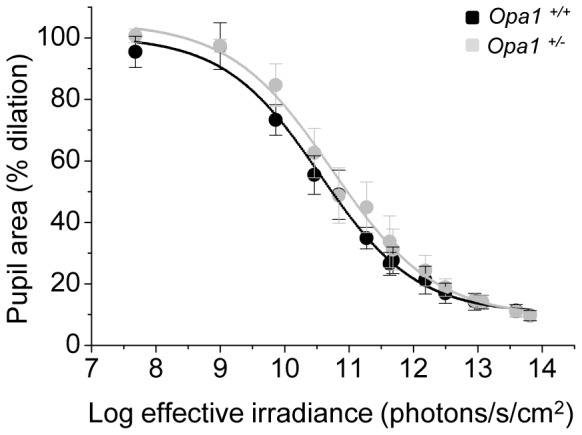
Pupil light reflex in *Opa1^+/+^* and *Opa1^+/−^* mice. The average minimum pupil area expressed as a percentage of maximum dilation following illumination with various intensities of white light for *Opa1^+/+^* (n = 5) and *Opa1^+/−^* (n = 5) mice. All data are fitted with four term sigmoidal functions (solid lines) of the form y = y0+a/(1+exp(-(x-x0)/b)) (goodness of fit of fitted curve to actual data (R2): *Opa1^+/+^* = 0.993 and *Opa1^+/−^* = 0.995). A 2-way ANOVA using intensity and genotype as factors showed a significant effect of light intensity (p<0.0001) but no significant effect of genotype (p = 0.51) and no significant interaction between genotype and intensity (p = 0.99).

Melanopsin-expressing and conventional RGCs contribute to the PLR [16], [17], [24], [34]. Both wildtype and *Opa1* mutant mice demonstrated a graded pupil constriction in response to increasing illumination across a full intensity range. The average irradiance response curve for *Opa1^+/−^* mice was very similar to that of wildtype littermates (Figure 5). A 2-way ANOVA using intensity and genotype as factors showed a significant effect of light intensity (p<0.0001) but no significant effect of genotype (p = 0.51) and no significant interaction between genotype and intensity (p = 0.99).

### *Opa1* defect has no effect on melanopsin expression

**Figure 6:**
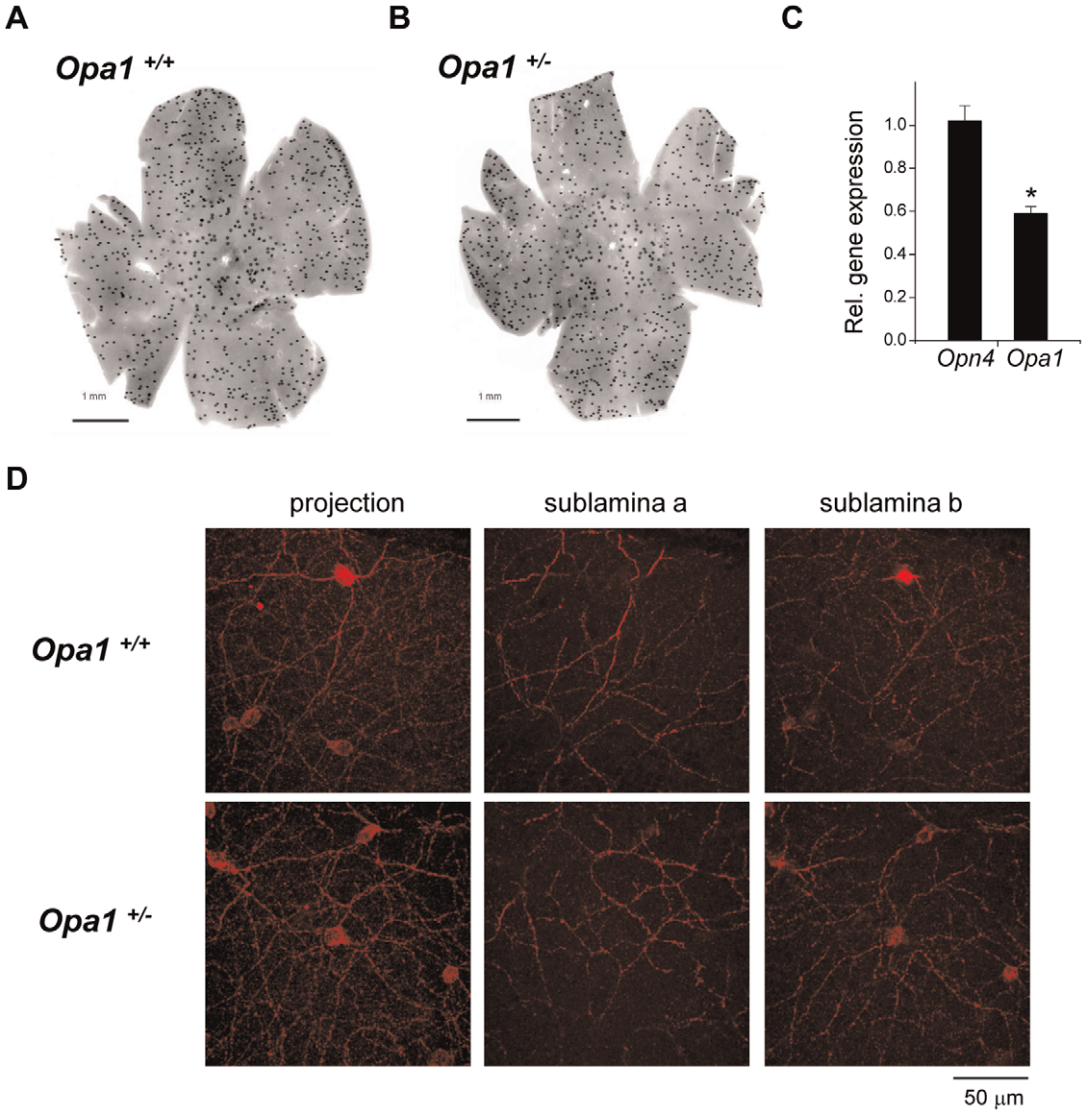
Melanopsin expression in *Opa1^+/+^* and *Opa1^+/−^* retinae. Overall distribution of melanopsin-positive RGCs in a flatmount retina from (A) *Opa1* ^+/+^ and (B) *Opa1* ^+/*−*^ mice. The total number of melanopsin expressing cells was not significantly different between genotypes (*Opa1^+/+^*: n = 3; *Opa1^+/^*: n = 3). (C) Quantification of melanopsin (*Opn4*) and *Opa1* gene expression by real time quantitative PCR. Expression levels in *Opa1^+/−^* animals are plotted relative to wildtype data. No significant difference in expression was detected for *Opn4* between genotypes. A significant reduction in *Opa1* expression was observed in *Opa1^+/−^* mice relative to wildtype controls (student's t-test. * = p<0.005). (D) Representative confocal images of melanopsin cells in *Opa1^+/+^* and *Opa1^+/−^* retinae. A projected image of a confocal stack (from the inner plexiform layer to the ganglion cell layer) is shown for each genotype. An image at the plane of the outermost region of sublamina a and an image at the plane of the innermost region of sublamina b from the same image stacks is also shown.

Melanopsin cells were assessed immunohistologically in retinal wholemounts (Figure 6A,B). There was no significant difference in the total number of melanopsin cells between genotypes (average cell count per retina: *Opa1* ^+/+^: 900±28.9 cells; *Opa1* ^+/*−*^: 810±37.9 cells). Morphological characterisation of the melanopsin cells also revealed no significant difference in the mean soma diameter (*Opa1* ^+/+^: 13.9±0.5 µm, n = 30; *Opa1* ^+/*−*^: 14.0±0.5 µm, n = 30; data not shown). Furthermore, we observed no obvious differences in the stratification patterns of the melanopsin cells between genotypes (Figure 6C). Melanopsin positive dendrites were observed in both sublamina a (OFF) and sublamina b (ON) of the innerplexiform layer. Finally, there was no significant difference in melanopsin transcript levels between *Opa1* ^+/+^ and *Opa1* ^+/*−*^ retinae (Figure 6D). As a control, we assessed *Opa1* transcript levels and found an ∼50% reduction in *Opa1^+/−^* retinae relative to wildtype controls_ (*p*<0.005) as expected[8], [9].

## Discussion

Abnormalities in the axons and dendritic arbors of RGCs in the B6; C3-*Opa1* ^Q285STOP^ mouse model of ADOA[8], [10] are believed to underlie functional visual dysfunction which begins to be apparent by 12 months of age [8], [12], [35]. Despite the pathophysiology of the RGC population, we have found that photic regulation of circadian activity, immobility-defined sleep behaviour and the pupil light reflex is preserved in *Opa1* mutant mice.

Patients with ADOA display optic nerve atrophy resulting in moderate to severe visual dysfunction [1], [3], [4]. However, these patients maintain a fully functional PLR[20], [21], suggestive of a preservation of NIF light driven functions. Due to the nature of circadian/sleep behavioural testing, other light driven NIF behaviours including circadian and sleep behaviours, have not been examined in patients. We performed a comprehensive assessment of NIF functions in a mouse model of ADOA. There was no significant difference in circadian photoentrainment, driven by melanopsin-expressing and conventional RGCs[16], [17], [18], between *Opa1* ^+/+^ and *Opa1* ^+/*−*^ mice. Negative masking, phase shift behaviour to 480 nm and 525 nm stimuli and acute light induction of sleep are likely to be entirely driven by intrinsically photosensitive melanopsin-expressing RGCs without significant input from conventional RGCs[16], [17], [18]. These behaviours were also preserved in *Opa1* mutant mice, suggesting preservation of melanopsin RGC derived responses. A previous study also assessed masking behaviour in the same *Opa1* mutant line[8] and suggested that masking in *Opa1* ^+/*−*^ mice was impaired relative to wildtype controls. It is likely that this discrepancy is due to the fact that the data were not corrected for individual differences in baseline activity. This may be important as we have noted that *Opa1* ^+/*−*^ animals display a slightly higher baseline activity level relative to *Opa1* ^+/+^ mice. In the present study this difference in baseline activity levels was not significant. The results overwhelmingly support a preservation of NIF light driven behaviour in *Opa1* mutant mice.

It has been previously reported that RGC soma loss is minimal in heterozygous B6; C3-*Opa1* ^Q285STOP^ mice[10]. In confirmation, we found no significant difference in melanopsin RGC numbers between wildtype and mutant mice. Hence, the lack of effect of the *Opa1* mutation on circadian function maybe due to the lack of a significant effect on melanopsin RGC cell numbers. However, previous studies have shown that visual function is disrupted in *Opa1^+/−^* mice [8], [12] despite the maintained numbers of RGCs. It was suggested that the visual disruption may be due to the observed RGC axonal abnormalities and the significant progressive RGC dendropathy [10]. Interestingly, RGC dendropathy was restricted to sublamina b of the inner plexiform layer (IPL). Melanopsin-expressing photosensitive RGCs stratify in sublamina a (M1 cells), sublamina b (M2, M4 and M5 cells) or in both sublaminae (M3) of the IPL[13], [36], [37], [38]. The different morphological melanopsin cell classes project to different brain centres and thus drive different NIF visual functions[13], [36]. The suprachiasmatic nucleus, which controls circadian behaviour, receives projections mainly from M1 cells[39], [40]. The olivary pretectal nucleus which drives the PLR, receives projections from both M1 and M2 cells[39], [40]. A specific deficit in the pupil light reflex may therefore be predicted in *Opa1* mutant mice. The pupil light reflex is driven by melanopsin at high irradiance[34]. At lower irradiances both rod and cone input to the melanopsin cells and conventional RGCs contribute to the PLR [16], [17], [24], [34]. Presently, a robust pupil light reflex was recorded in *Opa1* ^+/*−*^ mice, equivalent to wildtype controls, in response to a full irradiance series. This would suggest that all photoreceptor inputs to the PLR are preserved including dendritic inputs from the rod and cone pathways to the melanopsin RGCs. Immunohistological studies also revealed no apparent differences in melanopsin RGC dendritic projections to sublamina a and sublamina b of the IPL between *Opa1* ^+/+^ and *Opa1* ^+/*−*^ mice. These data would suggest that melanopsin RGCs are perhaps not subject to the dendritic reorganisation observed in other RGCs that stratify in sublamina b[10].

Several studies have suggested that the PLR is also preferentially preserved in another mitochondrial optic atrophy – Leber's hereditary optic neuropathy (LHON)[20], [41], [42], [43]. La Morgia et al. (2010) also reported a relative sparing of melanopsin RGCs relative to the total RGC population in ADOA and LHON patients[20]. Preservation of melanopsin ganglion cells has been reported in other models of retinal disease/damage[44], [45]. This may suggest that melanopsin RGCs are resistant in models of inner retinal damage, particularly in mitochondrial retinopathies.

It is unclear why melanopsin-expressing photosensitive RGCs may be less susceptible to mitochondrial impairment than other ganglion cells. It has been predicted that cells with a low energy demand may be affected least by loss of normal mitochondrial morphology or function. However, ultrastructural characterisation of the melanopsin cells has identified dendritic varicosities packed with mitochondria[46] arguing against this simple explanation. Melanopsin RGCs express the neuropeptide pituitary adenylate cyclise activating polypeptide (PACAP)[47]. Several studies have found that *in vivo* application of PACAP has retinoprotective effects[48], [49], [50]. However, it is unknown if melanopsin cells express PACAP receptors or if cellular expression of PACAP itself is neuroprotective in mitochondrial retinopathies.

We conclude that photic regulation of circadian behaviour, immobility-defined sleep induction and the pupil light reflex are preserved in a mouse model of ADOA despite previous reports of RGC pathophysiology[8], [10] and visual dysfunction[12], [35]. Melanopsin-expressing RGCs, which primarily drive these behaviours, may be resistant to damage in mitochondrial retinopathies. Identification of the possible neuroprotective mechanism involved could lead to exciting therapeutic strategies.
